# Impact of aprotinin and renal function on mortality: a retrospective single center analysis

**DOI:** 10.1186/1749-8090-6-103

**Published:** 2011-08-30

**Authors:** Brian Schloss, Parul Gulati, Lianbo Yu, Mahmoud Abdel-Rasoul, William O'Brien, Jon Von Visger, Hamdy Awad

**Affiliations:** 1 Department of Anesthesiology, The Ohio State University Medical Center, (410 West 10th Avenue), Columbus, (43210), USA; 2 Center for Biostatistics, The Ohio State University Medical Center, (2012 Kenny Road), Columbus, (43210), USA; 3 Perfusion Services, The Ohio State University Medical Center, (452 W. 10th Avenue), Columbus, (43210), USA; 4 Department of Nephrology, The Ohio State University Medical Center, (395 West 12th Avenue), Columbus, 43210, USA

**Keywords:** complex cardiac surgery, aprotinin, bleeding, renal dysfunction, mortality, antifibrinolytic drugs

## Abstract

**Background:**

An estimated up to 7% of high-risk cardiac surgery patients return to the operating room for bleeding. Aprotinin was used extensively as an antifibrinolytic agent in cardiac surgery patients for over 15 years and it showed efficacy in reducing bleeding. Aprotinin was removed from the market by the U.S. Food and Drug Administration after a large prospective, randomized clinical trial documented an increased mortality risk associated with the drug. Further debate arose when a meta-analysis of 211 randomized controlled trials showed no risk of renal failure or death associated with aprotinin. However, only patients with normal kidney function have been studied.

**Methods:**

In this study, we look at a single center clinical trial using patients with varying degrees of baseline kidney function to answer the question: Does aprotinin increase odds of death given varying levels of preoperative kidney dysfunction?

**Results:**

Based on our model, aprotinin use was associated with a 3.8-fold increase in odds of death one year later compared to no aprotinin use with p-value = 0.0018, regardless of level of preoperative kidney dysfunction after adjusting for other perioperative variables.

**Conclusions:**

Lessons learned from our experience using aprotinin in the perioperative setting as an antifibrinolytic during open cardiac surgery should guide us in testing future antifibrinolytic drugs for not only efficacy of preventing bleeding, but for overall safety to the whole organism using long-term clinical outcome studies, including those with varying degree of baseline kidney function.

## Background

Approximately one million cardiac surgeries are performed in the United States every year. Of these, about 200,000 can be classified as complex procedures, such as repeat coronary artery bypass grafting (CABG), valve replacements, and combined CABG with valve repairs/replacements. One of the reasons these procedures are labeled as complex is because they carry a significant increased risk of perioperative bleeding. An estimated 2.98% to 6.96% of high-risk cardiac operation patients return to the operating room due to bleeding [1]. Aprotinin (Bayer Pharmaceutical Corporation, West Haven, Connecticut), an antifibrinolytic agent, has been used extensively since a study showed that it reduced the need for blood transfusions during repeat cardiac surgery [2]. Since then, other clinical trials have confirmed aprotinin's efficacy in reducing the need for blood transfusions during these high-risk cardiac procedures [3,4].

The safety of aprotinin was brought into question in 2006 when a study revealed an increased risk of renal failure, myocardial infarction, and stroke [5]. In 2008, aprotinin was removed from the market after a large prospective, randomized clinical trial documented an increased mortality risk associated with the drug [6]. Further debate arose when a meta-analysis of 211 randomized controlled trials showed no increased risk of renal failure or death associated with aprotinin [4]. The ongoing debate about aprotinin's safety prompted us to examine clinical outcomes from our own institution that asked two specific questions: 1) What association does aprotinin have on all causes of mortality given varying levels of preoperative kidney dysfunction, and 2) What association does the drug have on all causes of mortality rates one year later in the same group of patients? Based on the previously mentioned studies, we proved that aprotinin increases the odds of death regardless of the level of a preoperative kidney dysfunction in these respective cohort patients.

## Methods

This retrospective, single-center study compared aprotinin versus no aprotinin use during complex cardiac surgery between October 2003 and October 2005. The study was conducted at The Ohio State University Medical Center in Columbus, Ohio. A total of 1,644 complex cardiac procedures were done during this two-year period, which included repeat CABG, valve replacements, and combined CABG with valve repairs/replacements. Non-complex cases, such as primary coronary artery bypass surgery, were specifically excluded in an attempt to avoid the statistical bias that aprotinin tends to be used in more complex surgeries, which inherently carry a greater morbidity and mortality risk [7]. The patient received the drug or no drug per the surgeons' request.

After obtaining approval from our institutional review board, we retrieved perioperative data from our institution's thoracic surgery, perfusion, and general electronic medical record databases. From the 1,644 cases, the Center for Biostatistics randomly selected 251 with varying degrees of renal dysfunction for analysis.

Twenty data points per patient were collected, including preoperative and postoperative kidney function, patient demographics, medical comorbidities, intraoperative variables, aprotinin administration, postoperative hemodialysis requirements, and one-year mortality (Table 1). Glomerular filtration rate, a measure for kidney function, was estimated using the Modification of Diet in Renal Disease study equation formula. This took into account serum creatinine measurement, age, sex, and race. The estimated glomerular filtration rate (eGFR) calculation was recorded in milliliters per minute. The decision to treat with aprotinin was based on surgeon preference. Follow-up data, including all causes of mortality at one year, were obtained using records from the electronic medical record database.

**Table 1:** Baseline Characteristics of Patients, According to Treatment Group

	No Aprotinin Mean (SD)	Aprotinin Mean (SD)	p-value
**Age**	61.1 (12.7)	64.5 (14.1)	0.045
**Height (cm)**	171.6 (8.9)	171.6 (10.6)	0.999
**Weight (kg)**	91.1 (22.9)	86.0 (20.5)	0.062
**Lowest HCT on bypass**	23.1 (4.8)	21.6 (4.7)	0.01
**Blood glucose (on bypass)**	231.6 (61.6)	245.8 (74.6)	0.10
**Blood glucose** **(48 hrs postoperative)**	202.1 (59.9)	216.5 (80.01)	0.11
**CPB duration**	75.9 (56.9)	113.8 (64.3)	< 0.001
**RBC transfusion on bypass**	1.27 (1.92)	2.13 (2.48)	
**Prime volume**	575.0 (205.2)	588.6 (324.3)	0.71
**UOP on pump**	261.5 (241.9)	287.3 (290.9)	0.51
	**No Aprotinin** **No. (%)**	**Aprotinin** **No. (%)**	**p-value**
**Male**	79 (68.1)	94 (70.15)	0.73
**Mild renal failure**	45 (38.79)	45 (33.33)	0.55
**Moderate renal failure**	39 (33.62)	45 (33.33)	
**Severe renal failure**	32 (27.59)	45 (33.33)	
**Diabetes**	49 (42.24)	51 (38.06)	0.50
**COPD none**	100 (86.21)	115 (85.19)	0.69
**COPD mild**	7 (6.0)	7 (5.2)	
**COPD mod**	8 (6.9)	9 (6.7)	
**COPD severe**	1 (0.9)	4 (3.0)	
**Hypertension**	90 (77.6)	110 (81.5)	0.44
**Diabetes**	49 (42.2)	51 (38)	
**Peripheral vascular disease**	17 (14.7)	28 (20.7)	0.21
**Myocardial infarction**	44 (37.9)	52 (38.5)	0.92
**Congestive heart failure**	42 (36.2)	83 (61.5)	< 0.001
**One year mortality**	20 (17.2)	43 (32.1)	0.007

COPD - chronic obstructive pulmonary disease; CPB - cardiopulmonary bypass; HCT - hematocrit; RBC - red blood cell; UOP - urine output

### Statistical analysis

Categorical demographic and clinical characteristics of patients were compared between the treatment and control groups using Chi-squared or Fisher exact tests as appropriate (Table 1). Continuous characteristics were compared between the treatment and control groups using Student *t *tests or Wilcoxon rank sum test where appropriate. The relationship between one-year mortality as the outcome variable and aprotinin treatment was analyzed using a multivariate logistic regression model that adjusted for other variables determined to be significantly related to mortality. Additional variables were checked for potential confounding or effect modification but did not make it into the final model as their effect on the relationship between aprotinin treatment and the outcome was minimal. We also adhered to the general guideline to include no more than one variable per 10 patients in the group that experienced the outcome event of interest. All statistical analyses were performed using SAS 9.2 (SAS, Carey, N.C.).

## Results

A total of 1,644 patients underwent complex cardiac surgery between 2003 and 2005. From this population, 251 were randomly selected for analysis. From this group, 39 were excluded from the final model because of incomplete data sets. A total of 212 subjects were included in our final statistical model.

Statistical analysis revealed three factors, other than aprotinin, that were significantly associated with mortality (Table 2). These factors were controlled in a multivariate logistic regression model. Based on this model, aprotinin use was associated with a 3.8-fold increase in odds of death compared to no aprotinin use (p = 0.0018) regardless of the level of preoperative kidney dysfunction after adjusting for other perioperative variables. The other three variables found to be significantly associated with death were diabetes, packed red blood cell transfusion on cardiopulmonary bypass, and 48-hour postoperative eGFR. In our model, diabetes was associated with a 2.2-fold increase in odds of death compared with non-diabetic patients (p = 0.0312) (Table 3). For packed red blood cell transfusion, the odds of death increased by 28% for every unit given while on bypass (p = 0.0018). Lastly, for every one unit (ml/min) increase in eGFR, the odds of death decreased by 2.4%.

**Table 2:** Multivariate Logistic Regression for One Year Postoperative Mortality in 184 Patients Showing Aprotinin Increased Death*

Effect	Odds Ratio (95% CI)	p-value
**Aprotinin vs. control**	3.830 (1.649, 8.893)	0.0018
**Diabetes**	2.236 (1.075, 4.651)	0.0312
**PRBC transfusion on bypass**	1.282 (1.096, 1.498)	0.0018
**eGFR 48 hours postoperatively**	0.976 (0.958, 0.994)	0.0079

CI - confidence interval; eGFR - estimated glomerular filtration rate; PRBC - packed red blood cells

* Excluded were 39 patients with missing values for at least one of the covariates in the model. The Hosmer-Lemeshow goodness of fit chi-square test statistic was 3.62 (P = 0.89).

**Table 3:** Results of Multivariate Logistic Regression for One Year Postoperative Mortality in 184 Patients*

Effect	Point Estimate	95% CI		p-value
**Aprotinin vs. control**	6.474	2.270	18.469	0.0005
**Diabetes vs. no Diab**	2.304	0.941	5.645	0.0679
**urine_output**	0.996	0.994	0.999	0.0019
**Prime_Vol**	1.002	1.000	1.003	0.0391
**Blood_Gluc_High_48_h**	1.007	1.000	1.013	0.0498
**RBC_trans**	1.422	1.176	1.720	0.0003
**Initial_Creatinine_m**	0.595	0.364	0.974	0.0388

CI - confidence interval; Gluc - glucose; RBC - red blood cell; Vol - volume

*Excluded were 67 patients with missing values for at least one of the covariates in the model. The Hosmer-Lemeshow goodness of fit chi-square test statistic was 3.62 (P = 0.89).

As expected, patients had varying levels of kidney dysfunction preoperatively, though level of kidney dysfunction did not differ significantly between the two groups. In our model, preoperative kidney function as a continuous or categorical variable did not significantly interact with aprotinin. This indicates that the increased odds of death in aprotinin-treated patients were the same across each level of kidney function.

The rates of postoperative hemodialysis were low for both drug and no drug groups. Only one patient in the no drug group and two patients in the aprotinin group required postoperative hemodialysis.

## Discussion

The primary new finding in our study is that aprotinin use, irrespective of the level of preoperative renal dysfunction, was associated with a 3.8-fold increase in odds ratio of death one year postoperatively. While previous studies have documented an increase in mortality associated with worsening preoperative kidney dysfunction [8,9], our model found no interaction between aprotinin and preoperative kidney dysfunction. Thus, our data supports the hypothesis that aprotinin use was associated with a 3.8-fold increase in odds of death compared to no aprotinin use (p = 0.0018) regardless of the level of a preoperative kidney dysfunction after adjusting for other perioperative variables.

This finding coincides with the Blood Conservation Using Antifibrinolytics in a Randomized Trial [6] and further supports the decision to remove aprotinin from the market. Our results support the opinion that the ability of aprotinin to reduce blood loss during complex cardiac surgery does not outweigh the risk of death associated with the drug. Furthermore, the continued availability of the lysine analogues, such as aminocaproic acid and tranexamic acid, lends little credibility to the continued use of aprotinin. Though there is no clear data showing the lysine analogues to be equally as efficacious as aprotinin [7,10], the risks of aprotinin likely do not outweigh the benefits.

There has been a certain degree of controversy over aprotinin's effect on kidney function postoperatively. Several previous retrospective studies have shown aprotinin to be associated with an increased risk of postoperative renal failure [11,12]. In contrast, the only prospective, randomized, placebo-controlled trial to investigate aprotinin's effects on kidney function postoperatively showed no significant difference between drug and control groups [13]. Furthermore, several retrospective studies have shown no increased incidence of dialysis in aprotinin-treated patients compared with control [14,15]. Our study showed no increase in hemodialysis in the aprotinin versus no aprotinin group. However, given the low rates of hemodialysis in both of our groups, it is possible that we missed a significant rise in risk of renal failure. It should also be noted that we were unable to obtain definitive causes of death during our data collection.

There were several other variables that were found to be significantly associated with mortality. We discuss these, as it was necessary to include them in our final statistical model. We found that the presence of diabetes, increased blood transfusions on bypass, and decreased eGFR all were associated with increased mortality. These findings are not surprising, and similar mortality associations have been demonstrated in the literature for diabetes [16-19] and red blood cell transfusion [20-25].

Our findings, while significant and new, have some limitations. The most significant of these limitations is the retrospective nature of the study. Also, the small number of deaths and our inability to document a cause of death may have potentially masked an increased risk of renal dysfunction as well as any other major organ system failures. In addition, our preoperative baseline of acute kidney dysfunction is non-standardized since there is no agreed upon definition of grades of acute renal dysfunction [26]. The aprotinin was administered to the patient per surgeon preference.

## Conclusions

The exact mechanism of increased mortality associated with aprotinin is unknown; however, it has been speculated that it may be related to its systemic effect on inflammation, oxidative stress, and bioactive molecules in addition to the complex interaction between the homeostatic, complement, and cytokine pathways in the setting of ischemia and reperfusion [27,28]. Ray and Stein [29] theorize that aprotinin's potential increase in mortality may simply be due to an over effectiveness of antifibrinolysis when compared to the lysine analogues. Perhaps the balance between procoagulatory and anticoagulatory effects is tipped too far in one direction by aprotinin. Novel pathways may exist that could be exerting deleterious affects on major organ systems. Regardless of speculation, it is clear that further research is necessary to elucidate the exact mechanisms that current and future antifibrinolytics exert on the whole organism, including long-term effects. A more complete understanding of their pathways will help to design drugs in the future that are as effective as they are safe. We believe that the lessons learned from our experience of using aprotinin as an antifibrinolytic during open heart surgery in the past should guide us in testing future drugs, which will not only focus on their efficacy of preventing bleeding, but on overall safety to the whole organism using long-term clinical outcome studies.

## List of Abbreviations

CABG: coronary artery bypass grafting; eGFR: estimated glomerular filtration rate

## Competing interests

The authors declare that they have no competing interests.

## Authors' contributions

BS analyzed the data and wrote the manuscript. PG, LY, MA, and WO analyzed the data. JV analyzed the data and wrote the manuscript. HA designed the study, analyzed the data and wrote the manuscript.

All authors read and approved the final manuscript.
